# Usability and performance expectancy govern spine surgeons’ use of a clinical decision support system for shared decision-making on the choice of treatment of common lumbar degenerative disorders

**DOI:** 10.3389/fdgth.2023.1225540

**Published:** 2023-08-15

**Authors:** Søren Eiskjær, Casper Friis Pedersen, Simon Toftgaard Skov, Mikkel Østerheden Andersen

**Affiliations:** ^1^ Department of Orthopedic Surgery, The Spine Research Group, Aalborg University Hospital, Aalborg, Denmark; ^2^ Department of Clinical Medicine, Aalborg University, Aalborg, Denmark; ^3^ Department of Orthopedic Surgery, Lillebaelt Hospital, Middelfart, Denmark; ^4^ Department of Orthopedic Surgery, University of Southern Denmark, Odense, Denmark; ^5^ Elective Surgery Center, Silkeborg Regional Hospital, Silkeborg, Denmark

**Keywords:** CDSS, UTAUT, PLS-SEM, usability, performance expectancy

## Abstract

**Study design:**

Quantitative survey study is the study design.

**Objectives:**

The study aims to develop a model for the factors that drive or impede the use of an artificial intelligence clinical decision support system (CDSS) called PROPOSE, which supports shared decision-making on the choice of treatment of ordinary spinal disorders.

**Methods:**

A total of 62 spine surgeons were asked to complete a questionnaire regarding their behavioral intention to use the CDSS after being introduced to PROPOSE. The model behind the questionnaire was the Unified Theory of Acceptance and Use of Technology. Data were analyzed using partial least squares structural equation modeling.

**Results:**

The degree of ease of use associated with the new technology (effort expectancy/usability) and the degree to which an individual believes that using a new technology will help them attain gains in job performance (performance expectancy) were the most important factors. Social influence and trust in the CDSS were other factors in the path model. *r*^2^ for the model was 0.63, indicating that almost two-thirds of the variance in the model was explained. The only significant effect in the multigroup analyses of path differences between two subgroups was for PROPOSE use and social influence (*p* = 0.01).

**Conclusion:**

Shared decision-making is essential to meet patient expectations in spine surgery. A trustworthy CDSS with ease of use and satisfactory predictive ability promoted by the leadership will stand the best chance of acceptance and bridging the communication gap between the surgeon and the patient.

## Introduction

Approximately 10,000 spinal surgeries are performed yearly in Denmark. Given that the patient-reported success rate for the outcome of spinal surgery 1 year postoperative is as low as 70–80%, there is room for improvement. Shared decision-making has been suggested to improve patient-reported outcomes of a given treatment (1, 2).

Shared decision-making is an approach where clinicians and patients share their knowledge, thoughts, preferences, and experiences about treatment before reaching a decision. While surgeons have a detailed knowledge about treatment options and the clinical evidence, uncertainties, benefits, and risks of each alternative, patients have in-depth information about their own everyday life, as well as their concerns, preferences, and goals when presented with the different options—the synthesis might very well be difficult (3).

Shared decision-making between the surgeon and the patient with a spinal disorder is often empirical and based on the surgeon's recent experience with a specific group of patients. However, it seldom encompasses all the unique characteristics of an individual patient. As a result, the decision to choose surgery may be severely biased. Even if we had absolute knowledge of all the variables influencing the outcome of spinal surgery for a particular patient, it might still be challenging to analyze and process these in the available time.

Predictive modeling using artificial intelligence (AI) and machine learning (ML) offers a solution for achieving more accurate predictive modeling of the outcome after spinal surgery. A search on prediction models and spine surgery yields 2,352 publications (PubMed), with a sharp increase in the number of publications from 2010 and onward.

We suggest that predictive modeling using AI or ML of the outcome of spinal surgery can aid in making the right treatment decision for a patient with spinal disorders. We have constructed a clinical decision support system (CDSS) named PROPOSE for that purpose. Based on patient-reported outcome measures (PROM), real-time predictions are generated for the outcome after surgery, including quality of life (EQ-5D, Oswestry Disability Index), back and leg pain, walking distance, return to work, and risk of complications.

However, several notable AI projects have failed. The most prominent was IBM Watson. In January 2022, the IBM Corporation sold Watson Health as it was not profitable (4). Benda et al. (5) pointed out that trust in AI is important and challenging, especially important with AI systems because explainability is low for these systems—the black box effect.

Several CDSS targeted at spine surgeons are available free of charge on the internet, e.g., Moulton et al. (6), Fritzell et al. (7), and Andersen et al. (8). However, the amount of actual use of these systems is probably very low in clinical practice. Almost no traffic was detected when measuring the traffic on the websites for the Dialogue Support System, in accordance with the literature on the subject (9).

The actual use of an information technology (IT) system depends on several factors; the two most fundamental are perceived usefulness/performance expectancy and perceived ease of use/effort expectancy (usability). Performance expectancy is “the degree to which an individual believes that using a new technology will help him or her to attain gains in job performance.” Effort expectancy is the “degree of ease of use associated with the new technology.” Social influence is also fundamental and is defined as “the degree to which an individual perceives the importance of how others believe that he or she should use the new technology.” The theoretical model used to describe the relationship between these factors (plus several more) and the behavioral intention and actual use is the Unified Theory of Acceptance and Use of Technology (UTAUT), first reported by Venkatesh et al. (10). In 2003, they developed the UTAUT model as a combination of several previous models from a range of disciplines. The goal is to explain technology acceptance to users. The original model consisted of four constructs, namely, performance expectancy, effort expectancy, social influence, and facilitating conditions. In 2012, the model was extended, with the UTAUT2 model directed at using consumer technologies. This model added hedonic motivation, price value, and habit to the original model. In the current context, we do not think hedonic motivation, price value, and habit are significant. Instead, we have added the composite variables trust, perceived risk, and resistance bias, which we suggest are much more meaningful, especially in the medical context. For a brief overview of the development of UTAUT models and criticism and advantages of the different models, we advocate going to https://acceptancelab.com/unified-theory-utaut.

This study aims to develop a model for the factors that drive or impede the use of an AI CDSS called PROPOSE, which supports shared decision-making on the choice of treatment of ordinary spinal disorders.

## Methods

**Figure 1:**
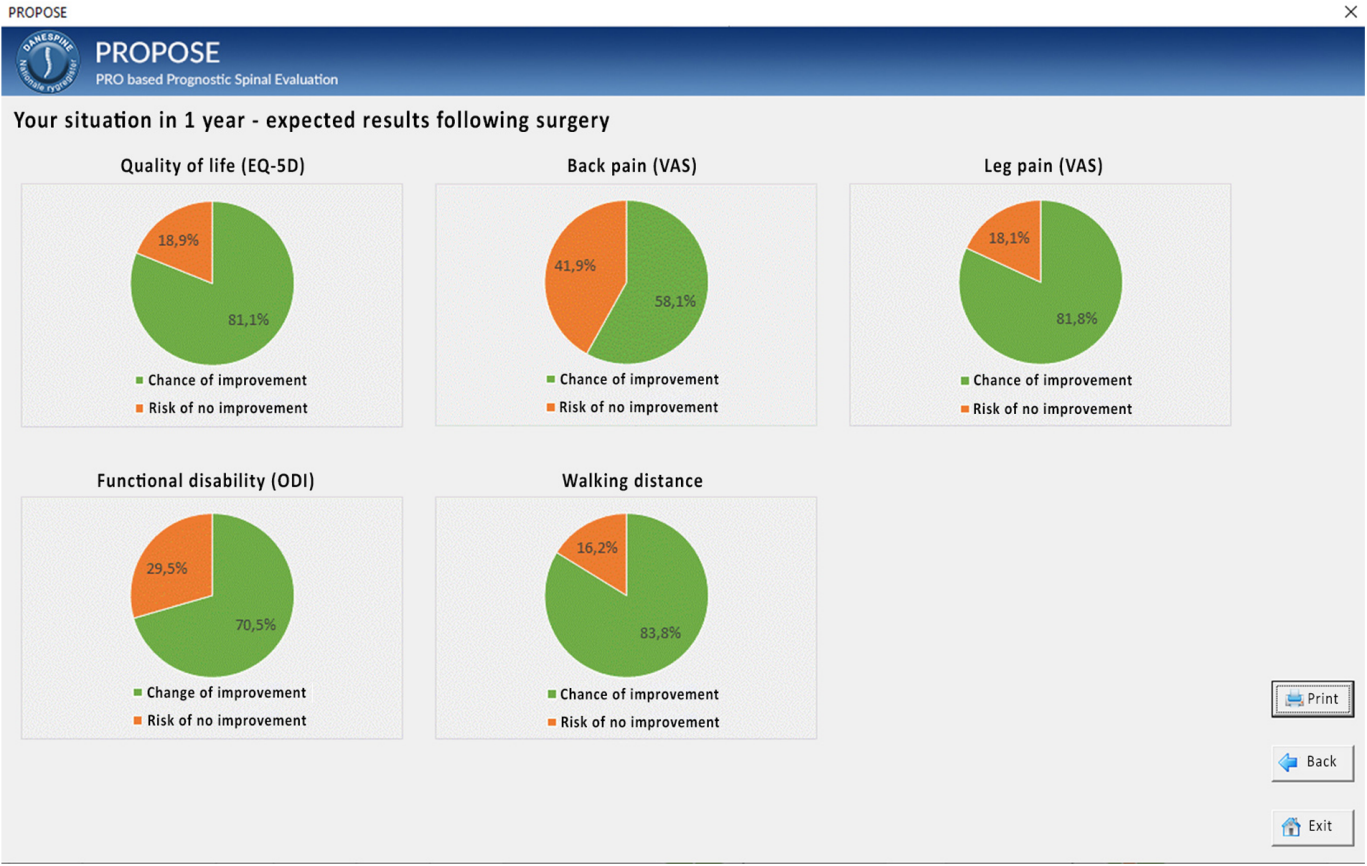
PROPOSE example: user interface for outcome after 1 year.

**Figure 2:**
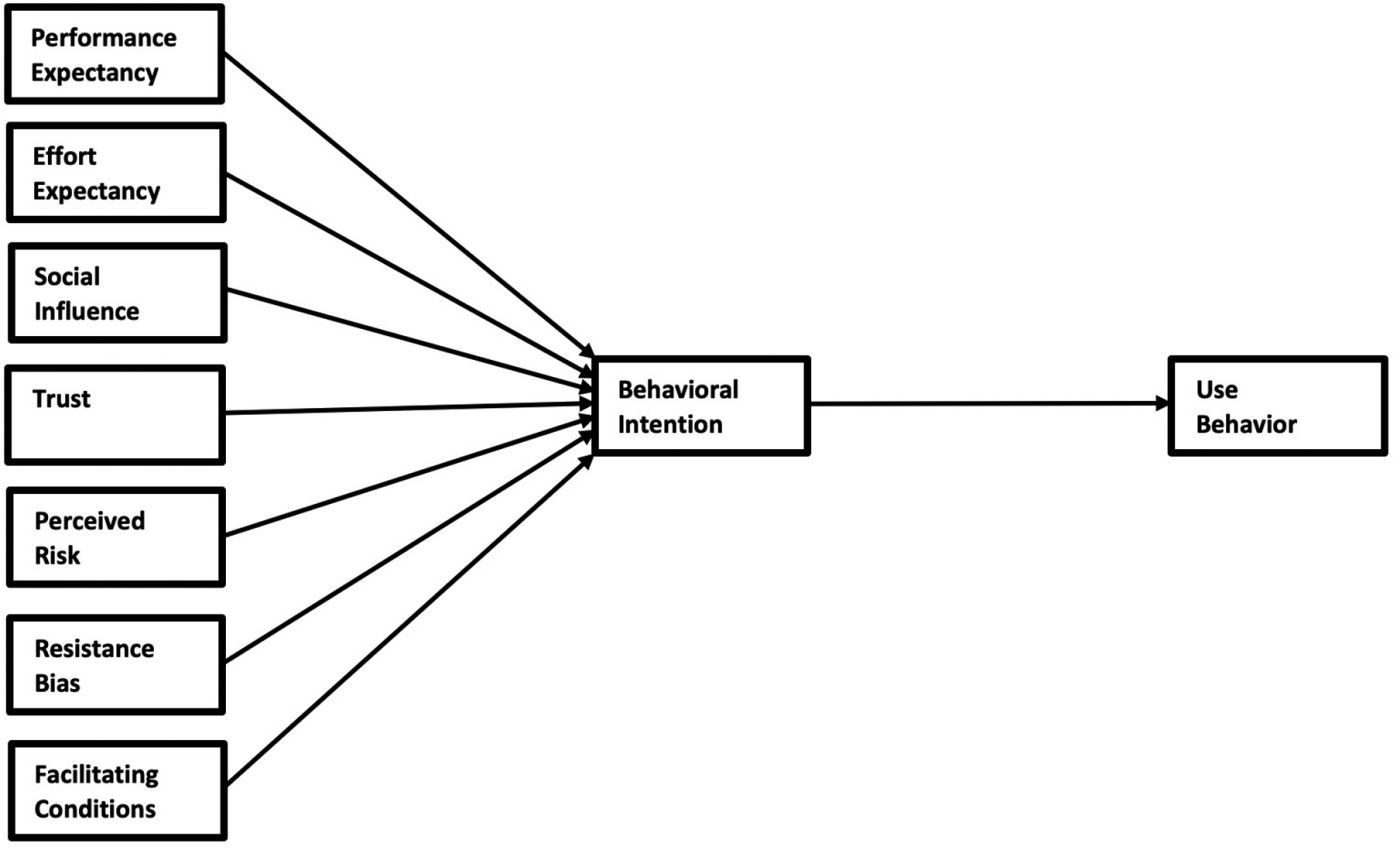
PROPOSE UTAUT preliminary model.

A web-based survey was opened to all participants of the Danish Spine Surgery Society (DRKS) and the Danish Orthopedic Society subspeciality meeting in the autumn of 2021. The questionnaire was based on the UTAUT model and extensions of this model. However, a number of questions concerning some demographic variables were also included. The questionnaire was distributed through a link to SurveyMonkey. A short PowerPoint presentation on PROPOSE (six slides) was made available for the participants as part of the survey, describing the system and showing details of the graphical user interface. All predictive models used in PROPOSE were constructed in R and R Studio using multivariate adaptive regression splines analysis, utilizing the packages “earth” and “caret.” Predictive models were implemented in a Microsoft Windows application coded in C# using Excel VBA. Before the meetings, PROPOSE had been used in three spine centers in Denmark, and some of the participants had used PROPOSE for some time, mainly in trial testing. All questions were obligatory and could be answered in 6–7 min. The seven-point Likert scale was used (from completely disagree to completely agree). The PowerPoint presentation and the Danish SurveyMonkey questionnaire are available as Supplementary Material. Figure 1 shows an example of the user interface. Data were exported as an Excel file (available as a Supplementary Material) and later imported into R study (the R code available as a Supplementary Material) for further analyses. Data were analyzed using partial least squares structural equation modeling (PLS-SEM). The minimum number of participants was calculated using the inverse square root method (11). The PLS-SEM approach usually requires fewer participants and can handle non-normal data and composite variables (composite variables are essential in this study). Based on previous findings from the literature (12), the minimum path coefficient was set to 0.35, power to 0.8, and significance level to 0.05. We then calculated the minimum sample size for our model to be *N* > 50. The detailed data analysis followed the outline reported by Hair et al. (13). The measurement models indicator reliability, internal consistency reliability, convergent validity, and discriminant validity were analyzed initially. In accordance with Hair et al. (14) (chapter 2, exhibit 2.9), a reflective measurement model was chosen as the most adequate. Figure 2 shows the preliminary model. Use behavior could not be assessed as only one-third of the participants had used PROPOSE, but this construct was analyzed in the multigroup analysis mentioned below. Table 1 lists the indicator variables reflecting the constructs and the scale statements/questions to be answered (Likert scale 1–7). The Danish questionnaire is available as a Supplementary Material. Table 2 demonstrates the *a priori* hypotheses. To assess indicator reliability, loadings above 0.7 were preferred, and all indicators with loadings below 0.4 were eliminated from the measurement model. The internal consistency reliability was assessed using the composite reliability rho_C_, Cronbach's alpha, and the reliability coefficient rho_A_. Convergent validity was evaluated using the average variance extracted (AVE). Alpha, rho_C_, and rho_A_ values should exceed 0.7, while the AVE value should exceed 0.5. The heterotrait–monotrait ratio (HTMT) was calculated to evaluate discriminant validity. HTMT values should be below 0.85. For the structural model, collinearity issues were analyzed by calculating the variance inflation factor (VIF) values. VIF values above 5 were considered indicative of collinearity issues among predictor constructs. The significance and relevance of the structural model relationship were assessed by applying bootstrapping. *t* values above 1.65 were considered statistically significant at the 10% significance level, which is commonly used in exploratory studies using PLS-SEM. The coefficient of determination (*r*^2^) was used to measure the explanatory power of the model. *r*^2^ values of 0.75, 0.50, and 0.25 were considered substantial, moderate, and weak, respectively. Multigroup analysis was undertaken using the variables age, gender, PROPOSE use, type of hospital, time in spine surgery, and personal innovativeness. The R package SEMinR (15) was the main package used for the data analysis—the R script is available as a Supplementary Material. Concerning ethics, participation was voluntary and anonymous and did not involve patients or any intervention. None of the participants received any financial reimbursement in relation to the survey. For that reason, no research ethics approval was necessary under Danish law.

**Table 1:** Variable items and scale statements.

	Scale statements
Performance expectancy	
PE1	I find PROPOSE useful in my job (9)
PE2	Using PROPOSE enables me to perform tasks quicker (11)
PE3	Using PROPOSE increases my productivity (10)
PE4	Using PROPOSE improves the outcome of my work (12)
Effort expectancy	
EE1	Propose is clear and understandable to me (13)
EE2	It was easy for me to become skillful at using PROPOSE (14)
EE3	I find Propose easy to use (15)
EE4	Learning to operate PROPOSE is easy for me (16)
Social influence	
SI1	People who influence my behavior think that I should use PROPOSE (17)
SI2	People who are important to me think that I should use PROPOSE (18)
SI3	In general, my hospital and department has supported the use of PROPOSE (19)
SI4	People whose opinion I value would like me to use PROPOSE (20)
Facilitating conditions	
FC1	I have the resources necessary to use PROPOSE (21)
FC2	I have the knowledge to use PROPOSE (22)
FC3	Health and IT personnel in the hospital are available to assist me with system difficulties (23)
FC4	I have adequate knowledge resources to help me learn about PROPOSE (24)
Behavioral intention	
BI1	I intend to use PROPOSE in the next 2 months (34)
BI2	I will use PROPOSE in the next 2 months (35)
BI3	I plan to use PROPOSE in the next 2 months (36)
Perceived risk	
PR1	There is a possibility of malfunction and performance failure, so PROPOSE might fail to deliver an accurate prognosis and could mislead my work with an inaccurate prognosis (45)
PR2	There is a probability that more time is needed to fix errors and nuances of the AI system PROPOSE (46)
PR3	I think using PROPOSE may cause psychological distress, as it could have a negative effect on my self-perception of the treatment plan (47)*
PR4	I am concerned that my patients’ personal information and health details are insecure and could be accessed by stakeholders or unauthorized persons leading to lawsuits for the physicians and the hospital (48)
Resistance bias	
RB1	I do not want PROPOSE to change how I develop my treatment plan because the new system is unfamiliar to me (49)
RB2	I do not want to use PROPOSE because of past experience; these new high-tech products always fall flat during practical application (50)
RB3	I do not want to use PROPOSE because there is a possibility of losing my job as AI-assisted technology may do the work better than me (51)
Use behavior	
UB1	I have already used PROPOSE (52)
UB2	I recommend others should use PROPOSE (53)
UB3	Have you ever overridden PROPOSE after using it for some time (54)
Personal innovativeness	
PI1	If I heard about a new technology, I would look for ways to experiment with it (38)
PI2	In general, I am among the first of my colleagues to acquire a new technology when it appears (39)
PI3	I like to experiment with new technologies (40)
Trust	
T1	I trust PROPOSE to be reliable (41)
T2	I trust PROPOSE to be secure (42)
T3	I believe clinical decision support systems like PROPOSE are trustworthy (43)
T4	I trust clinical decision support systems like PROPOSE (44)

**Table 2:** Hypotheses.

Hypothesis 1	Performance expectancy positively affects surgeons’ intention to use PROPOSE.
Hypothesis 2	Effort expectancy positively affects surgeons’ intention to use PROPOSE.
Hypothesis 3	Social influence positively affects surgeons’ intention to use PROPOSE.
Hypothesis 4	Trust positively affects surgeons’ intention to use PROPOSE.
Hypothesis 5	Perceived risk negatively affects surgeons’ intentions to use PROPOSE.
Hypothesis 6	Resistance bias negatively affects surgeons’ intentions to use PROPOSE.
Hypothesis 7	Facilitating conditions positively affects surgeons’ intentions to use PROPOSE.

## Results

**Figure 3:**
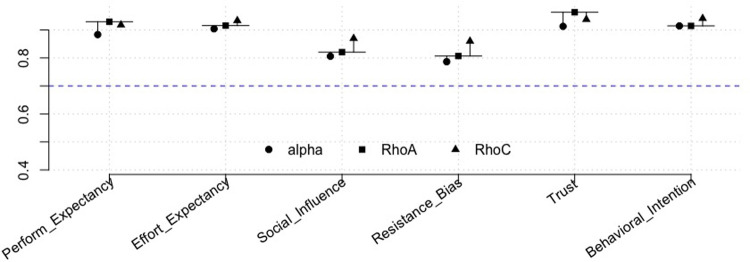
Internal consistency reliability.

**Figure 4:**
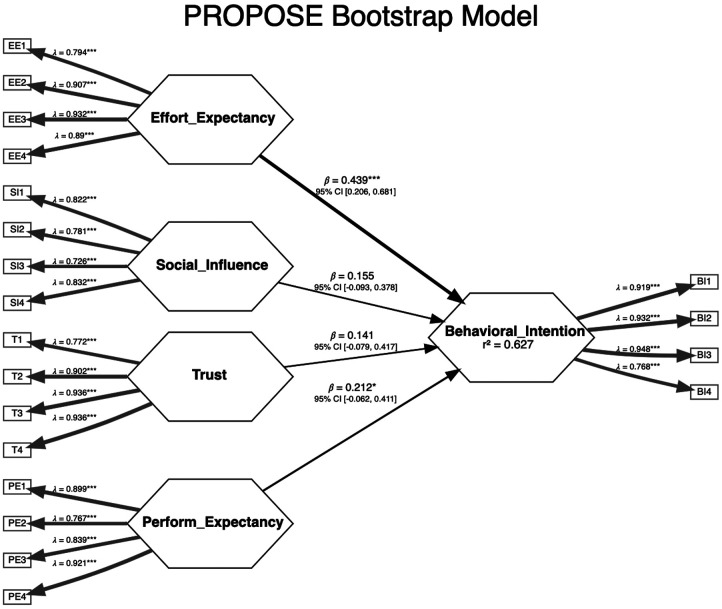
Final model—PROPOSE bootstrap model.

Table 3 shows the values of the demographic variables. All indicator loadings were above 0.7, except the indicator loading for the items (perceived risk) PR1, PR2, and PR4, which were all below 0.4 and, as a consequence, were eliminated from the model. PR3 had a loading above 0.7 and was retained. As the statement for the PR3 indicator showed significant similarity to the scale statements for resistance bias, we merged this into the indicators for the construct resistance bias as RB4. The construct “facilitating conditions” showed questionable path loadings for some of the indicators as well as questionable convergent and discriminant validity in the analysis, and we chose to exclude this from the analysis. It also seems reasonable to assume that facilitating conditions are less relevant for the simple app in question. Figure 3 demonstrates the values for alpha, rho_C_, and rho_A_. All AVE values exceeded 0.5 (0.60–0.80). All HTMT values were below 0.85. All VIF values were below 4. Figure 4 shows the evaluation of the structural model through bootstrapping for the final model. Only the path coefficients for effort and performance expectancy were significant. The *r*^2^ value for the model was 0.63—indicating that almost two-thirds of the variance in the model was explained. The adjusted *r*^2^ value was 0.6. Both values indicate moderate explainability. The only significant effect in the multigroup analyses of path differences between two subgroups was for PROPOSE use and social influence (*p* = 0.01). None of the other multigroup analyses with the variables mentioned above demonstrated significant path differences (significance level of 0.05).

**Table 3:** Demographics.

Variable	*N* = 62
Age	42 (23–69)^a^
Sex	
Male	53 (85%)
Female	9 (15%)
Type of hospital	
University	44 (71%)
Other	18 (29%)
Time in spine surgery	
1 year or less	27 (44%)
More than 1 year	35 (56%)
PROPOSE use	
Yes	19 (31%)
No	43 (69%)

^a^ Median, range.

## Discussion

We were able to confirm hypotheses 1 and 2. Effort expectancy or usability was the construct with the most significant influence on behavioral intention. Intentionally, we constructed the PROPOSE app with a careful choice of the number and type of variables, avoiding the need to enter the total score of PROM values and reducing the number of keystrokes to the absolute minimum. The additional time needed to complete PROPOSE was minimal. In addition, the user interface was intentionally simple and based on surgeon and patient input. Even the short PowerPoint presentation of the user interface seems to have conveyed the simplicity of the PROPOSE app.

The performance expectancy—the degree to which an individual believes that using a new technology will help them attain gains in job performance—also significantly influenced behavioral intention. The information about the important metrics of the prediction model was not incorporated into the information given to the survey participants as they were not available at the moment. The discrimination or calibration performance and the internal or external validation of the detailed underlying prediction model were not mentioned. However, it did mention that the prediction model had been constructed using AI and was based on the national Danish quality register for spine surgery (DaneSpine). The multigroup analysis did not disclose significant differences in the relationship between performance expectancy and behavioral intention for participants depending on age, experience, or any of the other variables mentioned above. This finding might indicate a perceived universal need for more than empiricism and gut feeling.

Social influence—the degree to which an individual perceives the importance of how others believe they should use the new technology—was insignificant in the bootstrapped path model, but its path coefficient value was the third largest. It is reasonable to assume that social influence can influence behavioral intention, given the right circumstances. This implies that if the leadership makes using a CDSS mandatory, at least the intention to use the DSS will improve. In fact, it has been demonstrated that the mandatory use of a CDSS improves healthcare quality (16). To further support this point, the only significant subgroup path difference was for PROPOSE use and social influence (source) and behavioral intention (target). Using the CDSS was almost mandatory for some time for those who had used PROPOSE. One cannot help but wonder why the mandatory use of a CDSS is undescribed in relation to spine surgery. Is this because the crucial model parameters are unknown to an extent where even the innovators themselves do not trust the CDSS or because they are unsure if the predictions are meaningful for the patients?

The group for which the use of PROPOSE was almost mandatory for a period of time was also the group with a higher number of experienced spine surgeons. The age of these experienced spine surgeons was also higher than the average age of all participants. A priori, we would expect younger surgeons to be more computer-literate and inclined to use a CDSS. Building on this postulate, we deduce that if social influence can impact the experienced more elderly surgeons, there is a probability that the group as a whole can be influenced to use a CDSS.

Trust and resistance bias are reciprocal entities. Both constructs had non-significant path loadings in the bootstrapped model. The coefficient for resistance bias had a negative sign and the smallest numeric value. The direction is as expected. The numeric value of the path component for trust was 1.7 times greater than that for resistance bias. Trust is an essential component in adopting a CDSS (17). Trust can be partitioned into benevolence belief (the CDSS acts in the interest of the clinician), integrity belief (the CDSS adheres to principles important to the clinicians), and competence belief (the CDSS can perform effectively) (9). The scale statements/questions for trust in our questionnaire probably do not reflect all three parts of trust. The statements mostly deal with competence belief, and we did not provide any data about the abilities, skills, and expertise of the CDSS as mentioned above. However, integrity belief could have been supported by the variables demonstrated in the user interface. Jansen-Kosterink et al. (9) found that benevolence and competence belief were the most important trust components. The participants had no actual knowledge of the competence of PROPOSE. They had none of the information required according to the TRIPOD (18) statement or PROBAST (19). Our advice is that, at a minimum, information about the number of patients, internal and external validation studies, discriminative ability, and calibration should be available on the website in question.

A repository for prediction models in spine surgery would be highly valuable. An alternative is doing a literature search and finding the link to the predictive model hidden somewhere in the text or Supplementary Material. A repository could also enforce a quality description for all predictive models it contains (20).

The indicator reliability for the scale statements/questions concerning perceived risk (except PR3) was unacceptable and had to be removed from the measurement model. This might reflect the quality of the questions and the adaptability to the specific situation since most participants had not used PROPOSE. In addition, PROPOSE does not give one specific unambiguous advice but rather a series of aspects or proposals to be discussed with the patient in the decision-sharing process—in all probability, this works to minimize the perception of any perceived risk. The PR3 question or indicator is concerned with the professional autonomy of the participants, which can be pinpointed as the central clinician characteristic affected by a CDSS (9).

The construct “facilitating conditions” was excluded because of a lack of convergent and discriminant validity. We suggest that the simple PROPOSE app and the intuitive user interface largely abolish the need for any assistance. However, in the case of a more complex CDSS, the construct “facilitating conditions” was the most important factor influencing behavioral intention (12).

One of the limitations of this study is the low number of participants. The number of surgeons doing spine surgery is limited in a country with 5.8 million inhabitants, and we did our very best to recruit participants, expecting that a person-to-person contact at the two abovementioned meetings would increase willingness to participate in the survey. Some of the participants had minimal knowledge and experience with spine surgery. However, this limitation is somewhat counteracted by the increase in age span and information technology ability. In addition, we could have incorporated other variables in the model, used another model, or posed the questions differently. The UTAUT model and its derivatives are well-tested models for these scenarios. The *r*^2^ values should be used with caution as it is a function of the number of predictor constructs—the adjusted *r*^2^ value compensates for this fact. According to the established guidelines, both *r*^2^ values can be characterized as moderate. Inventing a whole new model often results in low explainability. In the current scenario, other models such as the Fogg behavioral model could be of interest (21). The final important limitation is that most participants had not used PROPOSE and had to depend on a short presentation of the CDSS. This also means that the path between behavioral intention and use behavior could not be reviewed. However, usually, there is a strong correlation between behavioral intention and use behavior. It will be important to do a follow-up study incorporating both surgeon and patient opinion when PROPOSE is in full use.

Shared decision-making is extremely important to meet patient expectations; otherwise, some patients will be dissatisfied even when PROM values are improved significantly (22, 23).

We are supplying the usual goodness-of-fit (GOF) measures for the measurement and structural model but have not calculated any GOF for the total bootstrapped model. SEMinR currently cannot calculate GOF statistics for the total bootstrapped model. In the literature, an ongoing discussion on the relevance of GOF measures has not reached a definitive conclusion. We chose to adhere to the principles listed in one of our principal references on PLS-SEM (15), which is critical to using GOF measures. However, we are well aware that the use of GOF statistics is advocated by other researchers, including Schubert et al. (24). In addition, some members of the same group have pointed out that more research is needed to establish sound thresholds for these fit measures (25).

Shared decision-making using a CDSS with acceptable properties for the surgeon and the patients can fill some of the communication gaps. In conclusion, this study outlines the important properties of a CDSS that can enhance shared decision-making in spine surgery.

## Conclusion

Effort expectancy/usability and performance expectancy were found to be the most important and the only significant constructs influencing behavioral intention to use the CDSS named PROPOSE. The *r*^2^ value for the final bootstrapped model was moderate to substantial and certainly adds some credibility to the model. Though non-significant, there are indications that the construct “social influence” might improve the behavioral intention to use a CDSS. Improving trust/performance expectancy through detailed information on the internal and external validity of the CDSS should improve the behavioral intention to use a CDSS.

## Data Availability

The original contributions presented in the study are included in the article/**Supplementary Material**, further inquiries can be directed to the corresponding author.
