# Supplementary material for: Usability and performance expectancy govern spine surgeons’ use of a clinical decision support system for shared decision-making on the choice of treatment of common lumbar degenerative disorders
Source: Front Digit Health. 2023 Aug 15;5:1225540. doi: 10.3389/fdgth.2023.1225540 (PMC10465695; doi:10.3389/fdgth.2023.1225540)
Supplement: Supplementary file 2 [file Table2.docx]

# Model 1 #######################################################################

# Specifying the measurement model 1

UTAUT_PROPOSE_mm_1 <- constructs(

composite("Sex", single_item("Gender")),

composite("Age_in_Years", single_item("Age")),

composite("Use", single_item("PROPOSE_Use")),

composite("Hosp", single_item("Hospital")),

composite("Time", single_item("Time_in_Surg")),

composite("Personal_Innovativeness", multi_items("PI", 1:3)), #reflective

composite("Perform_Expectancy", multi_items("PE", 1:4)), #reflective

composite("Effort_Expectancy", multi_items("EE", 1:4)), #reflective

composite("Social_Influence", multi_items("SI", 1:4)), #reflective

composite("Perceived_Risk", multi_items("PR", 1:4)), #reflective

composite("Resistance_Bias", multi_items("RB", 1:3)), #reflective

composite("Facilitating_Conditions", multi_items("FC", 1:4)), #reflective

composite("Trust", multi_items("T", 1:3)), #reflective

composite("Behavioral_Intention", multi_items("BI", 1:4))) #reflective

# Specifying the structural model 1

UTAUT_PROPOSE_sm_1 <- relationships(

paths(from = c("Perform_Expectancy",

"Effort_Expectancy",

"Social_Influence",

"Perceived_Risk",

"Resistance_Bias",

"Facilitating_Conditions",

"Trust"), to =

"Behavioral_Intention"))

# Estimating the model 1

UTAUT_PROPOSE_modeling_1 <- estimate_pls(data = UTAUT_PROPOSE,

measurement_model = UTAUT_PROPOSE_mm_1,

structural_model = UTAUT_PROPOSE_sm_1,

inner_weights = path_weighting,

missing = mean_replacement, )

# Summarizing the model 1

Summary_UTAUT_PROPOSE_modeling_1 <- summary(UTAUT_PROPOSE_modeling_1)

Summary_UTAUT_PROPOSE_modeling_1$descriptives$statistics

Summary_UTAUT_PROPOSE_modeling_1$iterations

# Low number of iterations expected

Summary_UTAUT_PROPOSE_modeling_1$paths

# Facilitating_Conditions negative value

Summary_UTAUT_PROPOSE_modeling_1$reliability

# AVE > 0.5, Facilitating_Conditions and especially Perceived_Risk do not reach threshold values

plot(Summary_UTAUT_PROPOSE_modeling_1$reliability)

# values should be above blue line

Summary_UTAUT_PROPOSE_modeling_1$loadings

# loadings above 0.7 recommended but deleting indicators between 0.4 and 0.7 only if

# this leads to an increase in internal consistency reliability or convergent validity

# indicators with loadings below 0.4 should always be removed

# for PR1, PR2 and PR4 loading are below 0.4

# FC3 and FC4 below 0.7

Summary_UTAUT_PROPOSE_modeling_1$validity$htmt

# discriminant validity HTMT values < 0.85

# Facilitating_Conditions several values above 0.85

# Bootstraping the model

Boot_UTAUT_PROPOSE_modeling_1 <- bootstrap_model(seminr_model = UTAUT_PROPOSE_modeling_1,

nboot = 1000,

cores = NULL,

seed = 123)

# in final analysis nboot ≥ 10000

Sum_Boot_UTAUT_PROPOSE_modeling_1 <- summary(Boot_UTAUT_PROPOSE_modeling_1, alpha = 0.10)

Sum_Boot_UTAUT_PROPOSE_modeling_1$bootstrapped_HTMT

# upper boundaries should always be lower than 0.9

Sum_Boot_UTAUT_PROPOSE_modeling_1$bootstrapped_paths

Sum_Boot_UTAUT_PROPOSE_modeling_1$bootstrapped_loadings

Sum_Boot_UTAUT_PROPOSE_modeling_1$bootstrapped_HTMT

plot(Boot_UTAUT_PROPOSE_modeling_1, title = "Bootstrap Model")

# Model 2 ######################################################################

# Specifying the measurement model 2

UTAUT_PROPOSE_mm_2 <- constructs(

composite("Sex", single_item("Gender")),

composite("Age_in_Years", single_item("Age")),

composite("Use", single_item("PROPOSE_Use")),

composite("Hosp", single_item("Hospital")),

composite("Time", single_item("Time_in_Surg")),

composite("Personal_Innovativeness", multi_items("PI", 1:3)), # reflective

composite("Perform_Expectancy", multi_items("PE", 1:4)), # reflective

composite("Effort_Expectancy", multi_items("EE", 1:4)), # reflective

composite("Social_Influence", multi_items("SI", 1:4)), # reflective

composite("Resistance_Bias", multi_items("RB", 1:4)), # reflective

composite("Facilitating_Conditions", multi_items("FC", 1:4)), # reflective

composite("Trust", multi_items("T", 1:4)), # reflective

composite("Behavioral_Intention", multi_items("BI", 1:4))

) # reflective

# Specifying the structural model 2

UTAUT_PROPOSE_sm_2 <- relationships(

paths(from = c("Perform_Expectancy",

"Effort_Expectancy",

"Social_Influence",

#"Perceived_Risk",

"Resistance_Bias",

#"Facilitating_Conditions",

"Trust"), to =

c("Behavioral_Intention"))

)

# Facilitating Conditions of little value for a simple app.

# Only PR3 with loading values greater than 0.7 - very much like resistance bias and

# merged with RB as RB4 - copy of PR3

# Estimating the model 2

UTAUT_PROPOSE_modeling_2 <- estimate_pls(data = UTAUT_PROPOSE,

measurement_model = UTAUT_PROPOSE_mm_2,

structural_model = UTAUT_PROPOSE_sm_2,

inner_weights = path_weighting,

missing = mean_replacement, )

# Summarizing the model 2

Summary_UTAUT_PROPOSE_modeling_2 <- summary(UTAUT_PROPOSE_modeling_2)

Summary_UTAUT_PROPOSE_modeling_2$iterations

# lower than 300 iterations - max. number

# here the number of iterations is 6

Summary_UTAUT_PROPOSE_modeling_2$loadings

# loadings above 0.7 recommended but deleting indicators between 0.4 and 0.7 only if

# this leads to an increase in internal consistency reliability or convergent validity

# indicators with loadings below 0.4 should always be removed

# only PR3 was greater than 0.4 and is kept in the model as RB4 - see above

Summary_UTAUT_PROPOSE_modeling_2$reliability

# AVE > 0.5 and the others > 0.7

plot(Summary_UTAUT_PROPOSE_modeling_2$reliability)

# values should be above blue line

Summary_UTAUT_PROPOSE_modeling_2$descriptives$statistics

Summary_UTAUT_PROPOSE_modeling_2$paths

Summary_UTAUT_PROPOSE_modeling_2$validity$htmt

# discriminant validity HTMT values < 0.85

# Bootstraping the model - discriminant validity

Boot_UTAUT_PROPOSE_modeling_2 <- bootstrap_model(seminr_model = UTAUT_PROPOSE_modeling_2,

nboot = 10000,

cores = NULL,

seed = 123)

# nboot final ≥ 10000

Sum_Boot_UTAUT_PROPOSE_modeling_2 <- summary(Boot_UTAUT_PROPOSE_modeling_2, alpha = 0.10)

# For exploratory model alpha = 0.1 recommended

Sum_Boot_UTAUT_PROPOSE_modeling_2$bootstrapped_HTMT

Sum_Boot_UTAUT_PROPOSE_modeling_2$bootstrapped_paths

Sum_Boot_UTAUT_PROPOSE_modeling_2$bootstrapped_loadings

plot(Boot_UTAUT_PROPOSE_modeling_2, title = "PROPOSE Bootstrap Model")

summary(UTAUT_PROPOSE$Age)

summary(UTAUT_PROPOSE$PI1)

summary(UTAUT_PROPOSE$Time_in_Surg)

summary(UTAUT_PROPOSE$Hospital)

summary(UTAUT_PROPOSE$PROPOSE_Use)

summary(UTAUT_PROPOSE$Gender)

summary(UTAUT_PROPOSE$FC4)

####-----------EVALUATION MULTI GROUP ANALYSIS-----------####

UTAUT_PROPOSE_mga_Age <- estimate_pls_mga(UTAUT_PROPOSE_modeling_2, UTAUT_PROPOSE$Age < 42, nboot=1000, cores = 2)

summary(UTAUT_PROPOSE_mga_Age)

UTAUT_PROPOSE_mga_Age$source

UTAUT_PROPOSE_mga_Age$target

UTAUT_PROPOSE_mga_Age$estimate

UTAUT_PROPOSE_mga_Age$group1_beta

UTAUT_PROPOSE_mga_Age$group2_beta

UTAUT_PROPOSE_mga_Age$diff

UTAUT_PROPOSE_mga_Age$group1_beta_mean

UTAUT_PROPOSE_mga_Age$group2_beta_mean

UTAUT_PROPOSE_mga_Age$pls_mga_p

plot(UTAUT_PROPOSE_mga_Age)

UTAUT_PROPOSE_mga_PI1 <- estimate_pls_mga(UTAUT_PROPOSE_modeling_2, UTAUT_PROPOSE$PI1 < 5.5, nboot=1000, cores = 2)

summary(UTAUT_PROPOSE_mga_PI1)

UTAUT_PROPOSE_mga_PI1$source

UTAUT_PROPOSE_mga_PI1$target

UTAUT_PROPOSE_mga_PI1$estimate

UTAUT_PROPOSE_mga_PI1$group1_beta

UTAUT_PROPOSE_mga_PI1$group2_beta

UTAUT_PROPOSE_mga_PI1$diff

UTAUT_PROPOSE_mga_PI1$group1_beta_mean

UTAUT_PROPOSE_mga_PI1$group2_beta_mean

UTAUT_PROPOSE_mga_PI1$pls_mga_p

plot(UTAUT_PROPOSE_mga_PI1)

UTAUT_PROPOSE_mga_Use <- estimate_pls_mga(UTAUT_PROPOSE_modeling_2, UTAUT_PROPOSE$PROPOSE_Use < 2, nboot=1000, cores = 2)

summary(UTAUT_PROPOSE_mga_Use)

UTAUT_PROPOSE_mga_Use$source

UTAUT_PROPOSE_mga_Use$target

UTAUT_PROPOSE_mga_Use$estimate

UTAUT_PROPOSE_mga_Use$group1_beta

UTAUT_PROPOSE_mga_Use$group2_beta

UTAUT_PROPOSE_mga_Use$diff

UTAUT_PROPOSE_mga_Use$group1_beta_mean

UTAUT_PROPOSE_mga_Use$group2_beta_mean

UTAUT_PROPOSE_mga_Use$pls_mga_p

plot(UTAUT_PROPOSE_mga_Use)

# p = 0.014 for Social_Influence as source and target Behavioral_Intention

UTAUT_PROPOSE_mga_Time <- estimate_pls_mga(UTAUT_PROPOSE_modeling_2, UTAUT_PROPOSE$Time_in_Surg < 2, nboot=1000, cores = 2)

summary(UTAUT_PROPOSE_mga_Time)

UTAUT_PROPOSE_mga_Time$source

UTAUT_PROPOSE_mga_Time$target

UTAUT_PROPOSE_mga_Time$estimate

UTAUT_PROPOSE_mga_Time$group1_beta

UTAUT_PROPOSE_mga_Time$group2_beta

UTAUT_PROPOSE_mga_Time$diff

UTAUT_PROPOSE_mga_Time$group1_beta_mean

UTAUT_PROPOSE_mga_Time$group2_beta_mean

UTAUT_PROPOSE_mga_Time$pls_mga_p

plot(UTAUT_PROPOSE_mga_Time)

UTAUT_PROPOSE_mga_Hosp <- estimate_pls_mga(UTAUT_PROPOSE_modeling_2, UTAUT_PROPOSE$Hospital < 2, nboot=1000, cores = 2)

summary(UTAUT_PROPOSE_mga)

UTAUT_PROPOSE_mga_Hosp$source

UTAUT_PROPOSE_mga_Hosp$target

UTAUT_PROPOSE_mga_Hosp$estimate

UTAUT_PROPOSE_mga_Hosp$group1_beta

UTAUT_PROPOSE_mga_Hosp$group2_beta

UTAUT_PROPOSE_mga_Hosp$diff

UTAUT_PROPOSE_mga_Hosp$group1_beta_mean

UTAUT_PROPOSE_mga_Hosp$group2_beta_mean

UTAUT_PROPOSE_mga_Hosp$pls_mga_p

plot(UTAUT_PROPOSE_mga_Hosp)
