# Supplementary figures and images for: Usability and performance expectancy govern spine surgeons’ use of a clinical decision support system for shared decision-making on the choice of treatment of common lumbar degenerative disorders
Source: Front Digit Health. 2023 Aug 15;5:1225540. doi: 10.3389/fdgth.2023.1225540 (PMC10465695; doi:10.3389/fdgth.2023.1225540)

## Slide 1
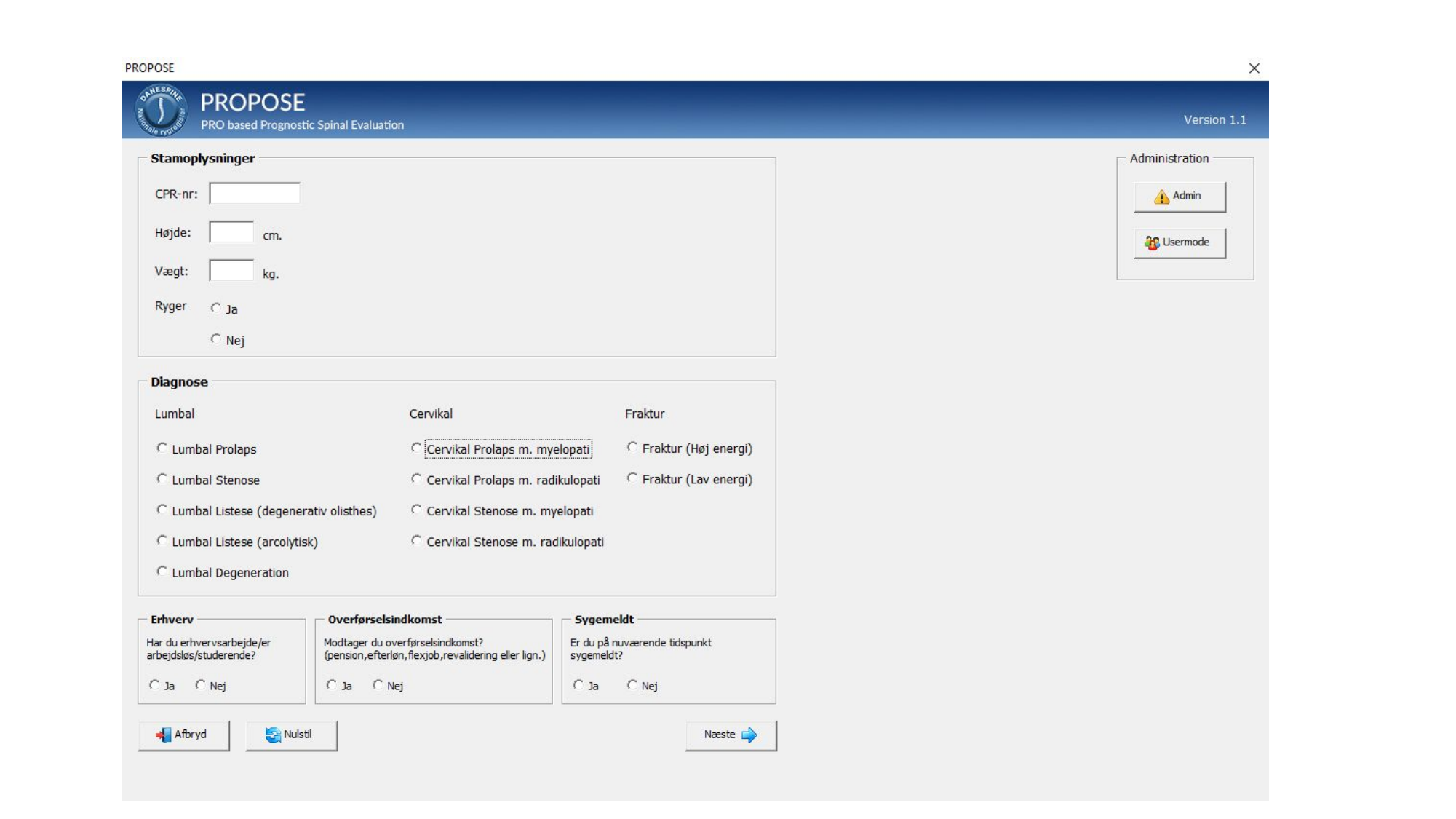

## Slide 2
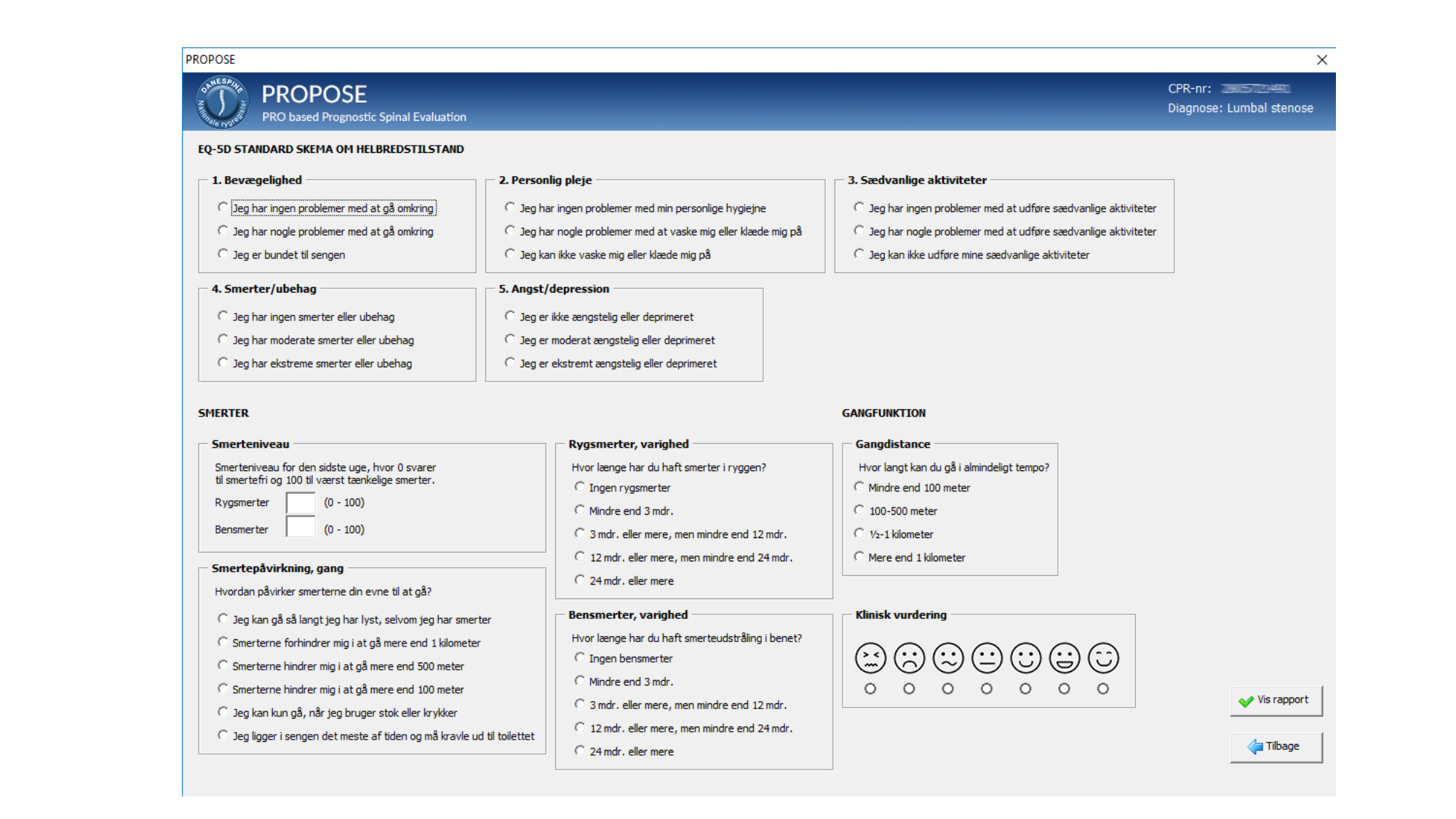

## Slide 3
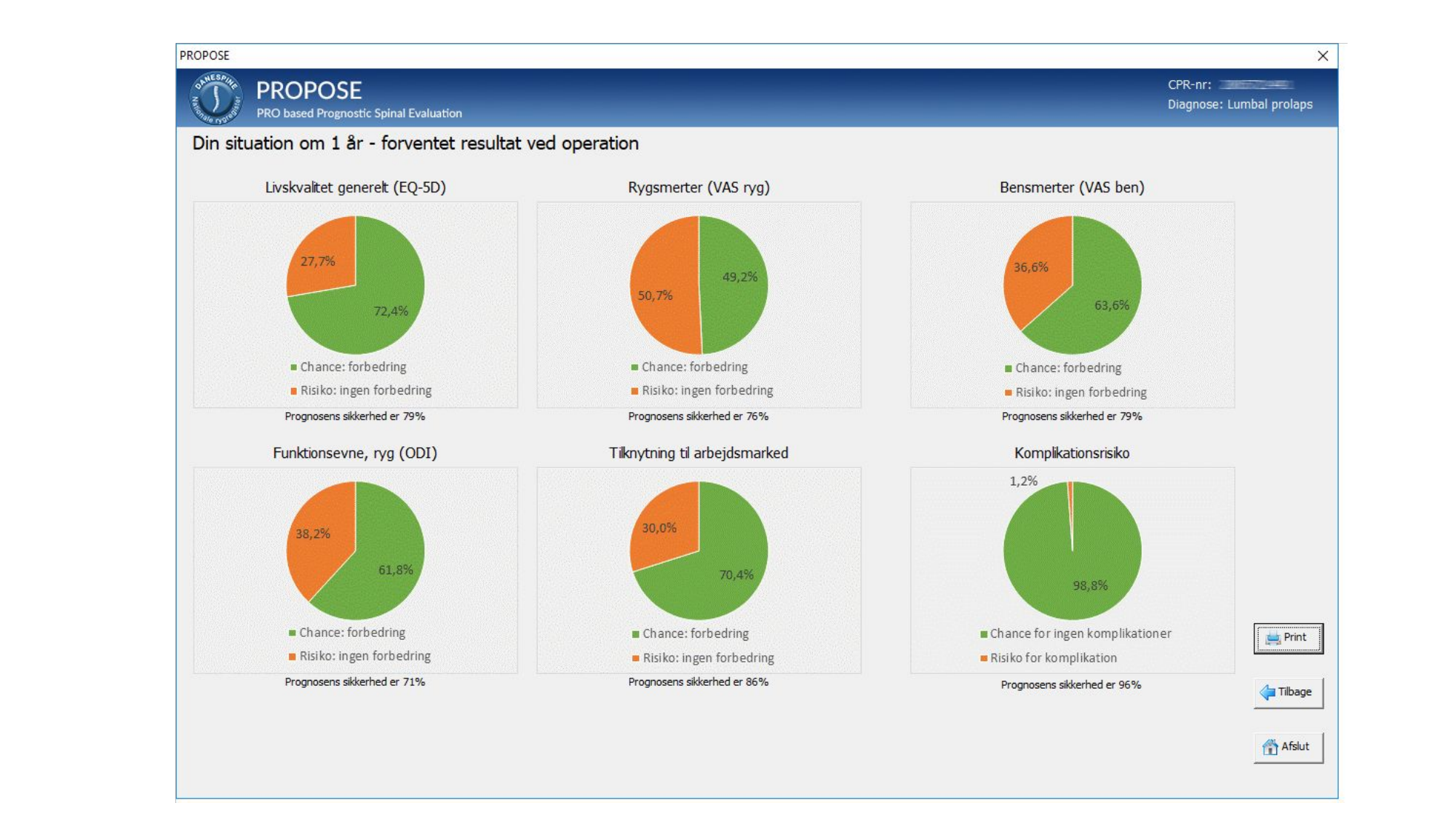

Supplement: Supplementary file 3 [file Presentation1.pptx]
