# Supplementary material for: Usability and performance expectancy govern spine surgeons’ use of a clinical decision support system for shared decision-making on the choice of treatment of common lumbar degenerative disorders
Source: Front Digit Health. 2023 Aug 15;5:1225540. doi: 10.3389/fdgth.2023.1225540 (PMC10465695; doi:10.3389/fdgth.2023.1225540)
Supplement: Supplementary file 4 [file Datasheet1.pdf]

PROPOSE spørgeskema

Information om PROPOSE

Den rygkirurgiske forskningsenhed ved Middelfart rygcenter har i et samarbejde med rygkirurger fra resten af Danmark udarbejdet et klinisk beslutningsstøttesystem (Clinical Decision Support System - CDSS) som kan anvendes i samtalen med patienten når der skal tages en behandlings-beslutning – stilles indikation for kirurgisk eller konservativ behandling. Beslutningsstøttesystemet er baseret på data fra DaneSpine den landsdækkende kliniske kvalitetsdatabase og er et IT-system som anvender Artificial Intelligence (AI). På baggrund af de tidligere registrerede data og den aktuelle patients individuelle data sandsynlig-gøres prognosen for en række parametre af betydning for patienten – smerte, livskvalitet (ODI), tilbagevenden til arbejdsmarkedet og komplikationsrisiko. Prognosen for disse parametre efter en rygkirurgisk operation præsenteres i den kliniske samtale med patienten og inddrager patienten i behandlings-beslutningen (shared decision making). Anvendelsen af et klinisk beslutningsstøtte-system er som alle andre IT-systemer helt afhængig af om brugeren er motiveret for at anvende systemet – det afhænger i princippet af brugerens opfattelse af i hvilken grad systemet vil forbedre brugerens arbejdssituation og af brugerens opfattelse af hvor let anvendeligt systemet er. Vi er meget interesseret i netop din opfattelse af et sådant system uanset om du har prøvet at anvende PROPOSE eller ej. Du vil få adgang til en kort PowerPoint præsentation om PROPOSE og en præsentation af en stor del af brugergrænsefladen. Spørgeskemaet bygger på en velevalueret teori om bruger accept af IT-systemer – Unified Theory of Acceptance and Use of Technology (UTAUT). Din vurdering vil medvirke til at vi kan øge systemets anvendelighed og du vil være blandt de første der får resultaterne af undersøgelsen. Du kan se PowerPoint præsentationen om PROPOSE og specielt brugergrænsefladen lige herunder eller på **https://www.dropbox.com/scl/fi/ke4kh9yxxe4ttes3njktt/PROPOSE.pptx?dl=0&rlkey=ndhavz2zelmuag9p83df4z2u8**

Slide 1

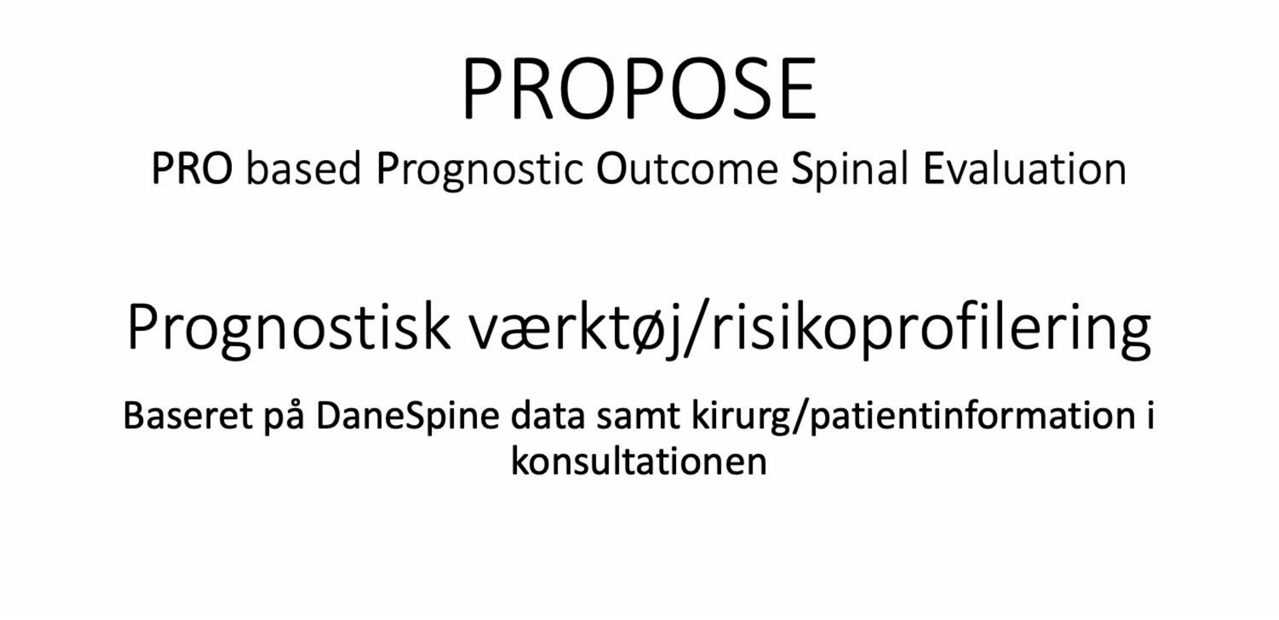

Slide 2

Virkemåde

1. Diagnose selekteres i værktøjet
2. I konsultationen registreres relevante basis-oplysninger ved afkrydsning/indtastning (eks. vægt, højde, rygestatus, sygemelding, smerter)
3. EuroQol-5 domæne spørgsmål i spørgeskemaform udfyldes
4. Ovennævnte oplysninger gemmes automatisk
5. På basis af DaneSpine data og de indhentede oplysninger genereres vha. (Artificial Intelligence – AI) algoritmer en prognose/risikoprofil
6. Resultatet præsenteres visuelt og gemmes i en fil med CPR-nr til evt. senere brug

Slide 3

Brugerinput

A). Brugerflade til afkrydsning/indtastning af oplysninger: Kun nødvendig information indsamles.

B). Spørgsmålene kan variere efter valgte diagnose.

C). Validering indbygget: der gøres opmærksom på manglende eller ikke valide svar.

Slide 4

Brugerinput: start

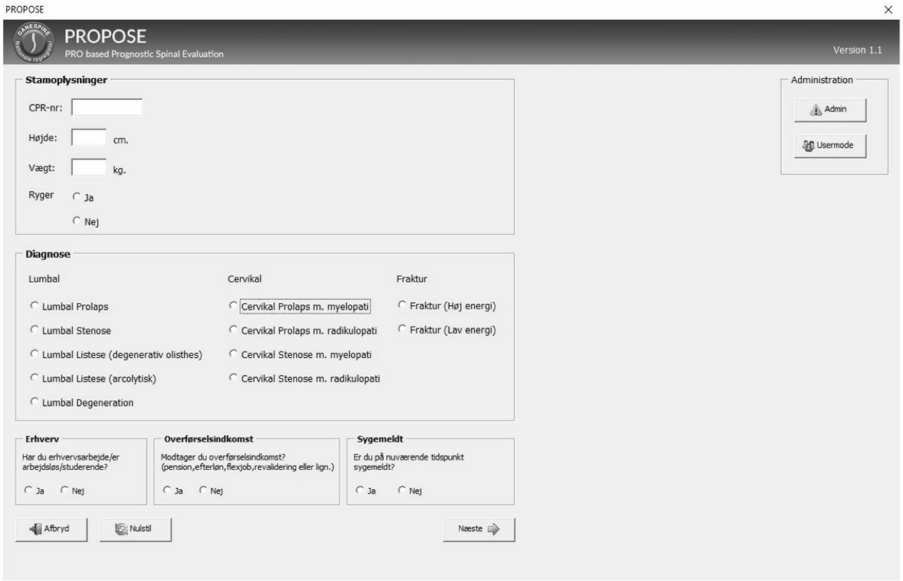

Slide 5

Brugerinput: spørgsmålsbesvarelse

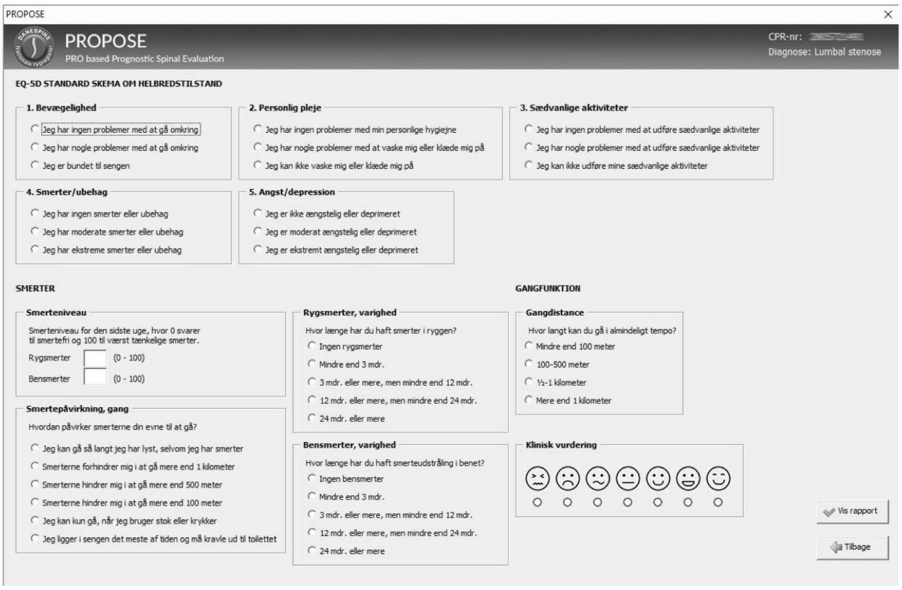

Slide 6

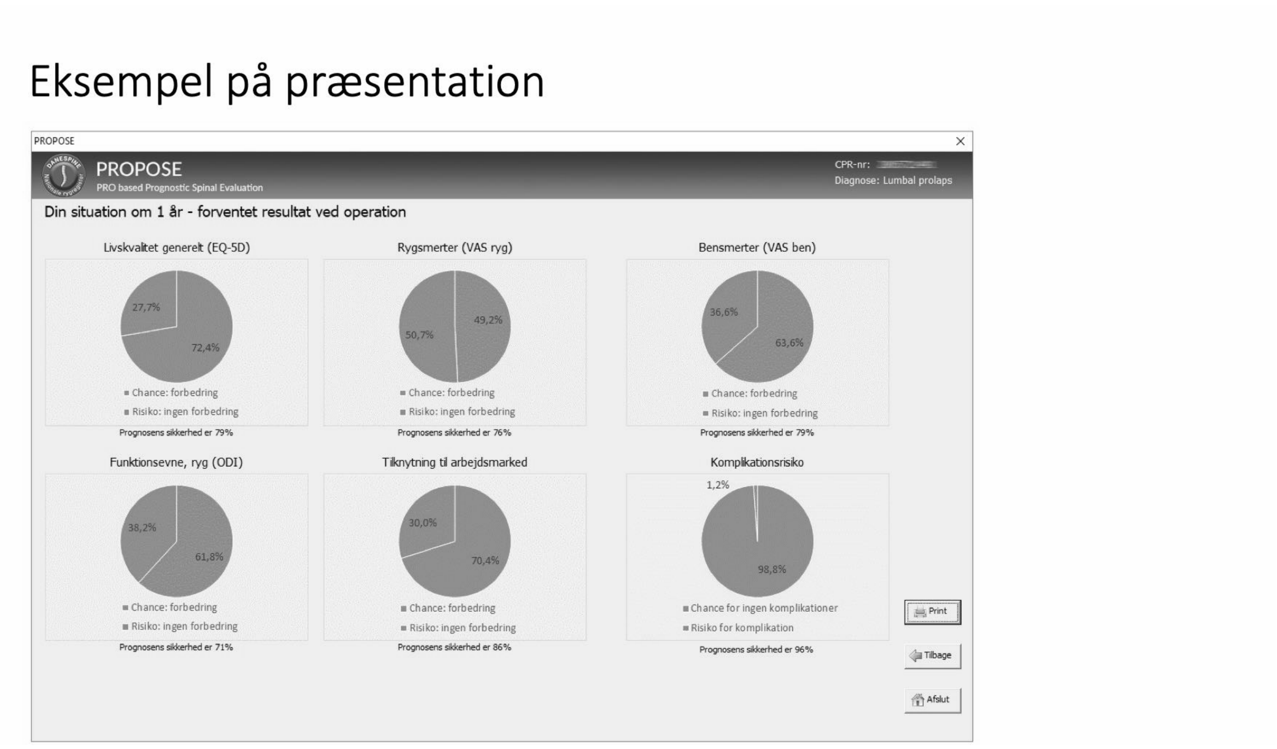

Demografika:

* 1. **Indtast venligst din E-mail-adresse:**

E-mail-adresse [ ]

* 2. **Indtast dags dato i formattet DD/MM/YYYY:**

Dato/klokkeslæt

Dato

DD/MM/ÅÅÅÅ

* 3. **Angiv din stillingsbetegnelse:**

- ○ Ledende overlæge
- ○ Specialeansvarlig overlæge
- ○ Afdelingslæge
- ○ 1. reservelæge eller reservelæge
- ○ Andet (angiv venligst)

[ ]

* 4. **Hvor lang tid har du beskæftiget dig med rygkirurgi:**

- Mindre end 1 år
- 1-4 år
- 5-10 år
- Mere end 10 år

* 5. **Angiv din alder i år:**

* 6. **Hvor er du ansat:**

- Universitetshospital
- Regionshospital
- Andet (angiv venligst)

* 7. **Hvilken del af rygkirurgien beskæftiger du dig hovedsageligt med:**

- Degenerative rygsygdomme
- Andet

* 8. **Har du allerede foretaget registrering i PROPOSE på dit arbejde :**

- Ja
- Nej

UTAUT Spørgsmål:

* 9. **PROPOSE vil være nyttig for mig i mit arbejde:**

- Meget uenig
- Uenig
- Lidt uenig
- Hverken uenig eller enig
- Lidt enig
- Enig
- Meget enig

* 10. **PROPOSE vil bevirke at jeg øger min produktivitet:**

- Meget uenig
- Uenig
- Lidt uenig
- Hverken uenig eller enig
- Lidt enig
- Enig
- Meget enig

* 11. **PROPOSE vil sætte mig istand til at udføre mit arbejde hurtigere:**

- Meget uenig
- Uenig
- Lidt uenig
- Hverken uenig eller enig
- Lidt enig
- Enig
- Meget enig

* 12. **PROPOSE vil medvirke til at resultatet af mit kliniske arbejde forbedres:**

- Meget uenig
- Uenig
- Lidt uenig
- Hverken uenig eller enig
- Lidt enig
- Enig
- Meget enig

* 13. **Min interaktion med PROPOSE vil være klar og let forståelig:**

- Meget uenig
- Uenig
- Lidt uenig
- Hverken uenig eller enig
- Lidt enig
- Enig
- Meget enig

* 14. **Det vil være let for mig at blive dygtig til at anvende PROPOSE:**

- Meget uenig
- Uenig
- Lidt uenig
- Hverken uenig eller enig
- Lidt enig
- Enig
- Meget enig

* 15. **PROPOSE vil være let at anvende:**

- Meget uenig
- Uenig
- Lidt uenig
- Hverken uenig eller enig
- Lidt enig
- Enig
- Meget enig

* 16. **At lære at bruge PROPOSE vil være let for mig:**

- Meget uenig
- Uenig
- Lidt uenig
- Hverken uenig eller enig
- Lidt enig
- Enig
- Meget enig

* 17. **Mennesker med indflydelse på mine holdninger synes jeg skal anvende PROPOSE:**

- Meget uenig
- Uenig
- Lidt uenig
- Hverken uenig eller enig
- Lidt enig
- Enig
- Meget enig

* 18. **Mennesker som har betydning for mig synes jeg skal anvende PROPOSE:**

- Meget uenig
- Uenig
- Lidt uenig
- Hverken uenig eller enig
- Lidt enig
- Enig
- Meget enig

* 19. **Mit hospital og min afdeling støtter brugen af PROPOSE:**

- Meget uenig
- Uenig
- Lidt uenig
- Hverken uenig eller enig
- Lidt enig
- Enig
- Meget enig

* 20. **Mennesker hvis mening jeg værdsætter ser gerne jeg anvender PROPOSE:**

- Meget uenig
- Uenig
- Lidt uenig
- Hverken uenig eller enig
- Lidt enig
- Enig
- Meget enig

* 21. **Jeg vil have adgang til de nødvendige ressourcer når jeg skal bruge PROPOSE:**

- Meget uenig
- Uenig
- Lidt uenig
- Hverken uenig eller enig
- Lidt enig
- Enig
- Meget enig

* 22. **Jeg har den nødvendige viden til at anvende PROPOSE:**

- Meget uenig
- Uenig
- Lidt uenig
- Hverken uenig eller enig
- Lidt enig
- Enig
- Meget enig

* 23. **Min IT afdeling vil hjælpe mig når der er problemer med PROPOSE:**

- Meget uenig
- Uenig
- Lidt uenig
- Hverken uenig eller enig
- Lidt enig
- Enig
- Meget enig

* 24. **Jeg har bøger, dokumenter og IT medarbejdere som vil hjælpe mig med at lære mere om PROPOSE:**

- Meget uenig
- Uenig
- Lidt uenig
- Hverken uenig eller enig
- Lidt enig
- Enig
- Meget enig

* 25. **PROPOSE er kompatibelt med andre systemer som jeg anvender:**

- Meget uenig
- Uenig
- Lidt uenig
- Hverken uenig eller enig
- Lidt enig
- Enig
- Meget enig

* 26. **Jeg mener PROPOSE vil passe godt ind i min måde at arbejde på:**

- Meget uenig
- Uenig
- Lidt uenig
- Hverken uenig eller enig
- Lidt enig
- Enig
- Meget enig

* 27. **At anvende PROPOSE er en god ide:**

- Meget uenig
- Uenig
- Lidt uenig
- Hverken uenig eller enig
- Lidt enig
- Enig
- Meget enig

* 28. **PROPOSE vil gøre mit arbejde mere interessant:**

- Meget uenig
- Uenig
- Lidt uenig
- Hverken uenig eller enig
- Lidt enig
- Enig
- Meget enig

* 29. **At bruge PROPOSE vil være sjovt:**

- Meget uenig
- Uenig
- Lidt uenig
- Hverken uenig eller enig
- Lidt enig
- Enig
- Meget enig

* 30. **Jeg vil gerne bruge PROPOSE:**

- Meget uenig
- Uenig
- Lidt uenig
- Hverken uenig eller enig
- Lidt enig
- Enig
- Meget enig

* 31. **Jeg er ængstelig ved at anvende PROPOSE:**

- Meget uenig
- Uenig
- Lidt uenig
- Hverken uenig eller enig
- Lidt enig
- Enig
- Meget enig

* 32. **Jeg frygter at miste en masse information ved et forkert tastetryk under brugen af PROPOSE:**

- Meget uenig
- Uenig
- Lidt uenig
- Hverken uenig eller enig
- Lidt enig
- Enig
- Meget enig

* 33. **Jeg vil tøve med at anvende PROPOSE af frygt for at lave fejltagelser som ikke kan rettes:**

- Meget uenig
- Uenig
- Lidt uenig
- Hverken uenig eller enig
- Lidt enig
- Enig
- Meget enig

* 34. **PROPOSE virker skræmmende på mig:**

- Meget uenig
- Uenig
- Lidt uenig
- Hverken uenig eller enig
- Lidt enig
- Enig
- Meget enig

* 35. **Det er min intention at tage PROPOSE i brug i løbet af de næste par måneder:**

- Meget uenig
- Uenig
- Lidt uenig
- Hverken uenig eller enig
- Lidt enig
- Enig
- Meget enig

* 36. **Jeg vil anvende PROPOSE i løbet af de næste par måneder:**

- Meget uenig
- Uenig
- Lidt uenig
- Hverken uenig eller enig
- Lidt enig
- Enig
- Meget enig

* 37. **Jeg har en plan om at anvende PROPOSE i løbet af de næste par måneder:**

- Meget uenig
- Uenig
- Lidt uenig
- Hverken uenig eller enig
- Lidt enig
- Enig
- Meget enig

* 38. **Når jeg hører om ny teknologi forsøger jeg at finde ud af hvordan jeg kan afprøve den:**

- Meget uenig
- Uenig
- Lidt uenig
- Hverken uenig eller enig
- Lidt enig
- Enig
- Meget enig

* 39. **Blandt mine kollegaer er jeg oftest den første som afprøver en ny teknologi:**

- Meget uenig
- Uenig
- Lidt uenig
- Hverken uenig eller enig
- Lidt enig
- Enig
- Meget enig

* 40. **Jeg synes det er spændende at afprøve nye teknologier:**

- Meget uenig
- Uenig
- Lidt uenig
- Hverken uenig eller enig
- Lidt enig
- Enig
- Meget enig

* 41. **Jeg har tillid til at kliniske beslutningsstøtte systemer som PROPOSE er pålidelige:**

- Meget uenig
- Uenig
- Lidt uenig
- Hverken uenig eller enig
- Lidt enig
- Enig
- Meget enig

* 42. **Jeg har tillid til at kliniske beslutningsstøtte systemer som PROPOSE er sikre systemer:**

- Meget uenig
- Uenig
- Lidt uenig
- Hverken uenig eller enig
- Lidt enig
- Enig
- Meget enig

* 43. **Jeg har tillid til at kliniske beslutningsstøtte systemer som PROPOSE er valide systemer:**

- Meget uenig
- Uenig
- Lidt uenig
- Hverken uenig eller enig
- Lidt enig
- Enig
- Meget enig

* 44. **Jeg har tillid til kliniske beslutningsstøtte systemer som PROPOSE:**

- Meget uenig
- Uenig
- Lidt uenig
- Hverken uenig eller enig
- Lidt enig
- Enig
- Meget enig

* 45. **Det er muligt at PROPOSE fungerer dårligt og ikke afgiver en korrekt prognose således at min rådgivning bliver misvisende:**

- Meget uenig
- Uenig
- Lidt uenig
- Hverken uenig eller enig
- Lidt enig
- Enig
- Meget enig

* 46. **Det er muligt at AI-systemet PROPOSE endnu ikke er fuldt udviklet og at der bør anvendes mere tid på fejlretning og nuancering af informationen:**

- Meget uenig
- Uenig
- Lidt uenig
- Hverken uenig eller enig
- Lidt enig
- Enig
- Meget enig

* 47. **Jeg tror at anvendelsen af PROPOSE i den kliniske samtale kan være psykisk stressende idet det måske vil have en negativ påvirkning på min egen opfattelse af behandlingsforslaget :**

- Meget uenig
- Uenig
- Lidt uenig
- Hverken uenig eller enig
- Lidt enig
- Enig
- Meget enig

* 48. **Jeg er bekymret for om mine patienters data er usikre og vil kunne tilgås af uautoriserede personer og evt. resultere i sagsanlæg etc. :**

- Meget uenig
- Uenig
- Lidt uenig
- Hverken uenig eller enig
- Lidt enig
- Enig
- Meget enig

* 49. **Jeg ønsker ikke at PROPOSE skal have nogen indflydelse på min behandlingsplan da systemet er uvant for mig:**

- Meget uenig
- Uenig
- Lidt uenig
- Hverken uenig eller enig
- Lidt enig
- Enig
- Meget enig

* 50. **Jeg ønsker ikke at anvende AI-systemet PROPOSE fordi jeg tidligere har oplevet at lignende high-tech løsninger altid falder helt til jorden når de skal anvendes i praksis:**

- Meget uenig
- Uenig
- Lidt uenig
- Hverken uenig eller enig
- Lidt enig
- Enig
- Meget enig

* 51. **Jeg ønsker ikke at anvende AI-systemet PROPOSE fordi det er muligt at systemet måske er bedre end mig til at udføre mit arbejde og jeg muligvis kunne miste mit arbejde af den grund:**

- Meget uenig
- Uenig
- Lidt uenig
- Hverken uenig eller enig
- Lidt enig
- Enig
- Meget enig

* 52. **Jeg har allerede benyttet PROPOSE:**

- Meget uenig
- Uenig
- Lidt uenig
- Hverken uenig eller enig
- Lidt enig
- Enig
- Meget enig

* 53. **Jeg vil anbefale andre at benytte PROPOSE:**

- Meget uenig
- Uenig
- Lidt uenig
- Hverken uenig eller enig
- Lidt enig
- Enig
- Meget enig

* 54. **Har du efter at have anvendt systemet i nogen tid ind imellem valgt at underkende anbefalingerne fra PROPOSE:**

- Meget uenig
- Uenig
- Lidt uenig
- Hverken uenig eller enig
- Lidt enig
- Enig
- Meget enig

Tusind tak for din besvarelse vi vender tilbage med de foreløbige resultater i løbet af de næste uger.
